# Sustainable colorimetric/luminescent sensors enabled by armored lipid nanoparticles

**DOI:** 10.1186/s40580-022-00335-5

**Published:** 2022-09-30

**Authors:** Jinkyu Roh, Yong Ho Cho, Dong June Ahn

**Affiliations:** 1 Department of Chemical and Biological Engineering, Korea University, Seoul, 02841 Republic of South Korea; 2 KU-KIST Graduate School of Converging Science and Technology, Korea University, Seoul, 02841 Republic of South Korea

**Keywords:** Sustainable colorimetric sensing, Polydiacetylene nanoliposome, Silica armor, Layer-by-layer deposition, Size-selectivity

## Abstract

In this study, we developed a highly stable polymeric vesicle using a nanosilica-armor membrane to achieve a sustainable colorimetric/luminescent response. The silica armor can be grown directly as ~ 5 nm spherical nanoparticles on the surface of the diacetylene (DA) vesicle with liposomal structure. This can be accomplished via the modified Stöber reaction in pure water on a layer of amine linkers deposited on the vesicles. Once formed, the structural stability of the DA vesicles dramatically increased and remained so even in a dried powder form that could be stored for a period of approximately 6 months. Then, redispersed in water, the armored vesicles did not agglomerate because of the electric charge of the silica armor. After polymerization, the polydiacetylene (PDA) vesicles maintained an average of 87.4% their sensing capabilities compared to unstored vesicles. Furthermore, the silica membrane thickness can be controlled by reiteration of the electrostatic layer-by-layer approach and the direct hydrolysis of silica. As the number of silica armor membranes increases, the passage of the stimuli passing through the membranes becomes longer. Consequently, three layers of silica armor gave the PDA vesicles size-selective recognition to filter out external stimuli. These discoveries are expected to have large-scale effects in the chemo- and biosensor fields by applying protective layers to organic nanomaterials.

**Graphical Abstract:**

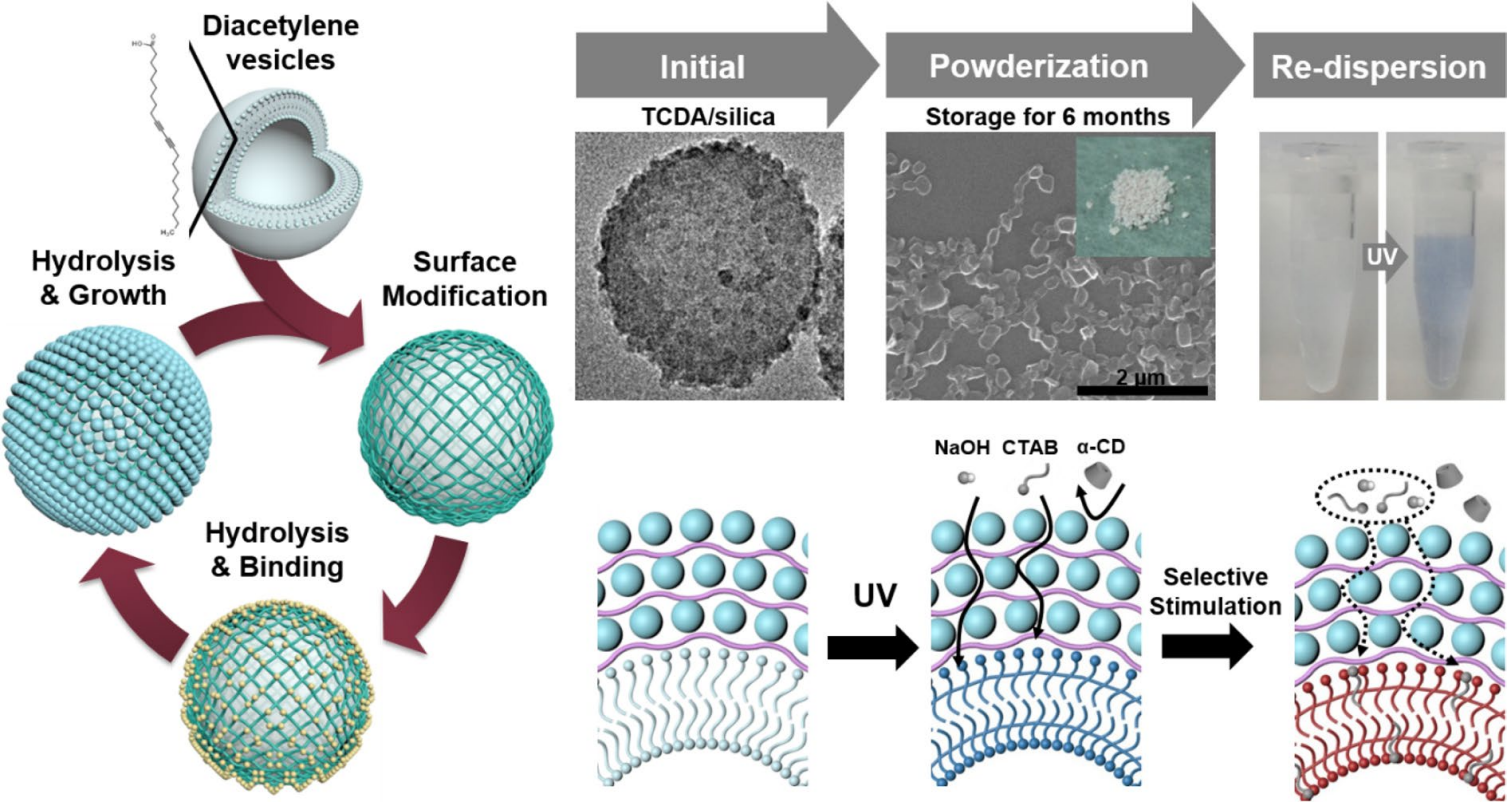

**Supplementary Information:**

The online version contains supplementary material available at 10.1186/s40580-022-00335-5.

## Introduction

Conjugated polymers have unique optical and electrical properties owing to proliferated delocalization of electrons in π-orbitals [1–3]. The optical properties of conjugated polymers are well known to vary with environmental stimuli and this characteristic has been used in sensor design [2, 3]. In the sensor research field, polydiacetylene (PDA) has been extensively investigated for decades owing to certain technical advantages including generation of strong “turn-on” optical responses to stimuli, methods of assembly available for transformation into various shapes, and rational molecular design enabling high specificity for desired targets [4–11]. However, the surfactant-based structure of diacetylene (DA) supramolecules has not been sufficiently stable for practical application in different fields [12, 13]. Like other organic materials, DA supramolecules can collapse and further aggregate over time to form bulk structures. After polymerization, PDA supramolecules are also susceptible to environmental factors, often triggering phase transitions to the stimulated state [4, 5]. These phenomena result in limited efficacy of PDA-based sensors. Therefore, the critical challenge is to maintain their functionality even after a long period of time, which can significantly impact DA-based technologies and their field applications.

To overcome these limitations, we devised a silica armor membrane that was placed directly on the surface of the liposomal structure of DA. Owing to its chemo-mechanical and thermal stability [14], biological compatibility [15], and permeability, silica is an important material that has been studied for the development of armored layers on living cells [16–18], fluorescent materials [19, 20], catalysts [21]. A few examples of adopting silica with PDA materials have been reported in connection with PDA-shell/silica-core composites for signal intensification [22, 23] purposes and in diacetylene–silane hybrid lamellas for reversibility control [24]. However, forming a layer of silica nanoparticles to cover the soft and delicate DA vesicles like an armor, thus endowing them with long-term stability, has never been investigated to date.

**Scheme 1:**
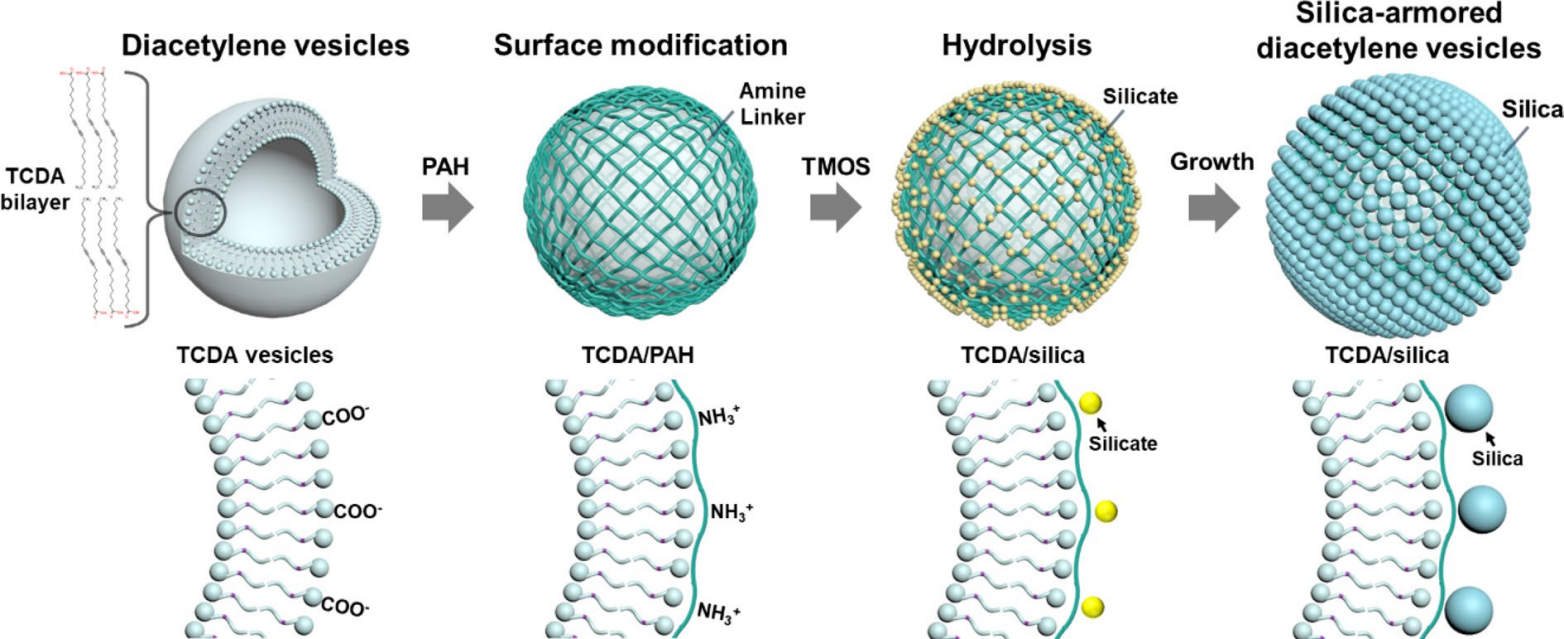
Synthesis method for silica-armored diacetylene vesicles

We first developed a novel process involving silica chemistry based on pure water and used a layer-by-layer (LbL) coating technique that was applied directly to the surfaces of the DA vesicular particles. Here, the silica nanoparticles grew on the vesicle surface by stober method [25] and acted as an armor layer to enhance its stability**,** as indicated in Scheme 1. We observed that this type of rigid silica armor protected the soft DA vesicles from harsh environmental conditions for extended storage periods and sustained their colorimetric response. Furthermore, existing pores among the silica nanoparticles provide passages for analytes to reach and stimulate polymerized DA vesicles within the armor, yielding optical responses. These pores can be controlled using an electrostatic LbL approach [26, 27] and the direct hydrolysis of silica, which can form continuous dense silica nanoparticles. By repeating this process, porous membranes of varying thicknesses can be formed on vesicles. The membrane permeability depends on its thickness; and we developed a size-selective recognition function for PDA vesicles with different stimulating molecules/structures.

## Methods

### Synthesis of TCDA vesicles

10,12-tricosadiynoic acid (1 mM, TCDA, GFS Chemical, 97%) was dissolved in chloroform (2 mL, anhydrous, Sigma-Aldrich, 99%). Then, the solution was filtered through a 200 nm pore syringe filter (Macherey–Nagel). The solution was then dried under N_2_ gas. Next, deionized water (20 mL) was added to the dried sample, which was heated to 80 °C and sonicated at 10 mW (Sonic Dismembrator 500, Fisher Scientific) for 15 min. This solution was then filtered using an 800 nm pore syringe filter (Merck Millipore) and stored at 4 °C for 6 h.

### Formation of silica armor on TCDA vesicles

To immobilize the amine groups on the surface of the TCDA vesicles, poly(allylamine hydrochloride) (0.1 mg/mL, PAH, Sigma-Aldrich) was mixed with the TDCA vesicles in deionized water for 30 min. The TCDA vesicles were then washed several times with deionized water. Tetramethylorthosilicate (100 mM, TMOS, purum, 98% (GC), Sigma-Aldrich) was added to pure water (pH 5.8) for 1 h and washed several times with deionized water. By repeating this method, a continuous silica armor membrane was formed on the surface.

### Confirmation of redispersibility

Powderized silica-armored TCDA vesicles (TCDA/silica) were redispersed in deionized water by sonication for 15 min. The redispersed TCDA/silica was polymerized using 254 nm UV light with a power density of 1 mW/cm^2^ (LF-215.S, UVItec) for 15 min.

### Reactivity tests by thermal and chemical stimuli

The redispersed TCDA/silica vesicles were stimulated by thermal stress at ~ 110 °C (WON-105, Daihan Scientific) for 15 min. Chemical stress was induced using alpha-cyclodextrin (10 mM, α-CD, USP standard, Sigma-Aldrich) and cetyltrimethylammonium bromide (10 mM, CTAB, BioUltra, > 99.0% (AT), Sigma-Aldrich) in deionized water and reacted for 1 h.

### Size-selective response of TCDA vesicles in silica armor membranes

TCDA vesicles with silica armor membranes were polymerized by exposure to 254 nm ultraviolet (UV) light (1 mW/cm2; LF-215.S, UVItec) for 10 min. Subsequently, various stimuli, including 10 mM NaOH (reagent grade, > 98%, pellets (anhydrous), Sigma-Aldrich), CTAB (Sigma-Aldrich), α-CD (Sigma-Aldrich), and γ-CD (powder, BioReagent, > 98%, Sigma-Aldrich) in deionized water were added to the vesicles. After reacting for 10 min, the optical responses of the stimulated samples were analyzed.

### Characterization

The morphology and chemical profile were observed using transmission electron microscopy (TEM), energy-dispersive X-ray spectroscopy (EDS, G2 F30, TECNAI), scanning electron microscopy (SEM, S-4300, Hitachi), and Fourier transform infrared spectroscopy (FT-IR, GX1, Perkin-Elmer). The optical properties were measured using UV–Vis spectroscopy (8453, Agilent), fluorescence spectroscopy (F-7000, Hitachi), and Raman spectroscopy (LabRam ARAMIS IR2, HORIBA JOBIN YVON). The surface charge and hydrodynamic diameter (HDD) were analyzed using electrophoretic light scattering spectroscopy (ELS-Z2, Otsuka Electronics). Finally, the pore sizes and surface areas were measured using an automatic gas and vapor adsorption analyzer (BELSORP-max, BEL Japan, Inc.).

## Results and discussion

10,12-Tricosadiynoic acid (TCDA) is an amphiphilic structure containing carboxylic acid groups and a long carbon tail that imparts a strong negative surface charge to vesicles (Additional file 1: Figure S1). To immobilize the silica armor on the vesicles, the vesicle surfaces must be modified with amines, which act as linkers between the vesicles and armor. Therefore, in this study, polyallylamine hydrochloride (PAH) was used as the linker and immobilized on the vesicles. PAH is an ionic polymer containing amine groups that can induce strong ionic bonds between the carboxylate functionalities of the vesicles and the silicate [28, 29]. Next, the growth of the silica on the surface must satisfy the silica hydrolysis condition while maintaining the vesicle stability. Typically, silica precursors are hydrolyzed in alcohol under both acidic and basic aqueous conditions; however, TCDA-based vesicular structures, which are framed with hydrogen bonds and hydrophobic interactions, are easily broken down under both acidic and basic conditions [13, 30, 31]. Therefore, we devised a method for the hydrolysis of silica precursors in pure water at pH ~ 5.8. Under these conditions, tetramethylorthosilicate (TMOS), a silicon alkoxide precursor, can be quickly hydrolyzed to silicate and, finally, can form silica nanoparticles while maintaining the monomeric TCDA vesicle structure.

**Fig. 1:**
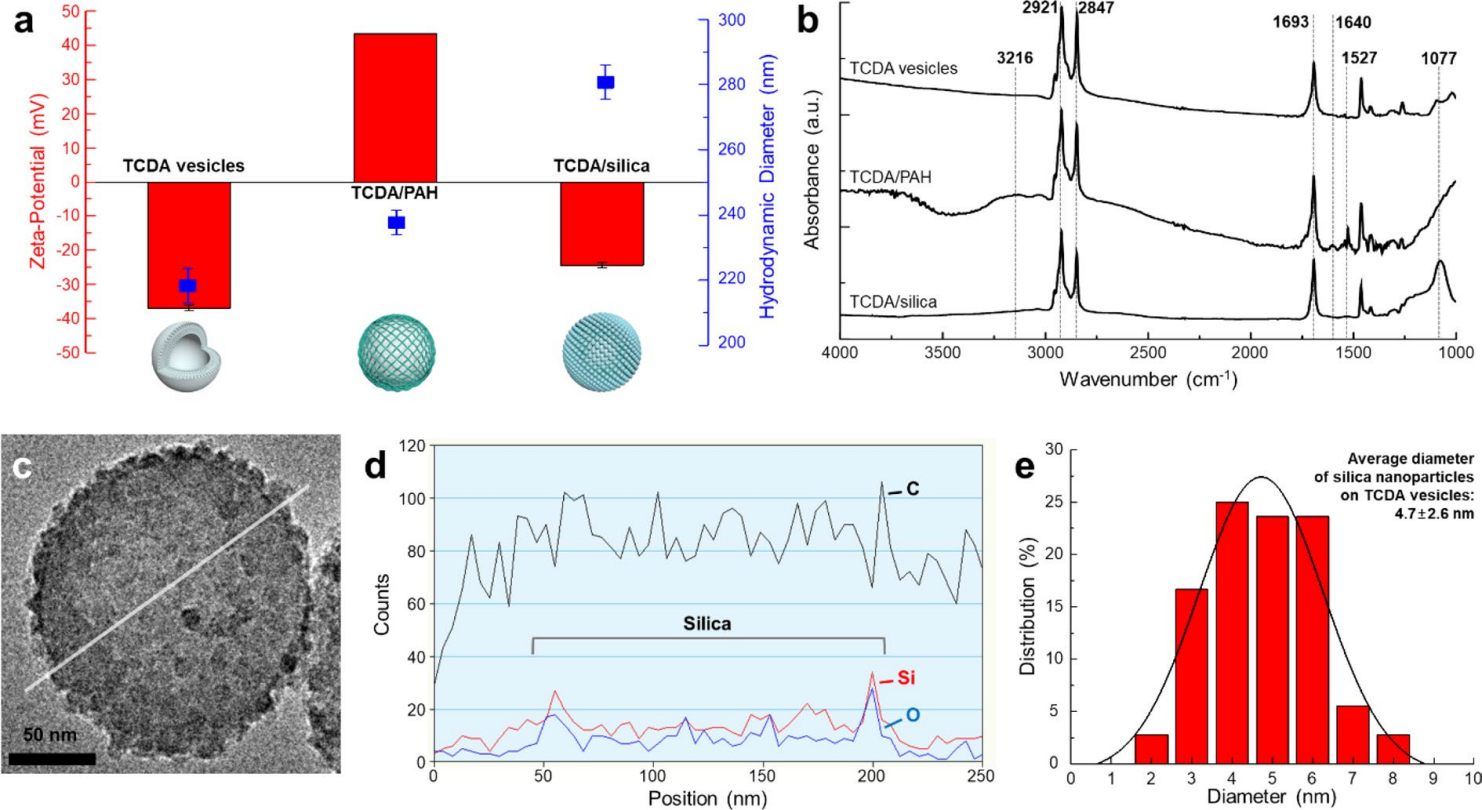
**a** Surface charge (left axis) and hydrodynamic diameter (HDD, right axis), and **b** Fourier transform infrared spectroscopy (FTIR) spectra of 10,12-Tricosadiynoic acid (TCDA) vesicles with different surface layers. **c** Transmission electron microscopy (TEM) image of monomeric TCDA/silica and **d** Energy dispersive spectroscopy (EDS) spectra of section shown by the white line in **c**. **e** Size distribution of silica nanoparticles on the TCDA vesicles

The surface charge of monomeric TCDA vesicles changed distinctly at every step with the immobilization of the layer: − 36 mV for the TCDA vesicles, + 43 mV for the PAH-immobilized TCDA vesicles (TCDA/PAH), and − 24 mV for the silica-armored TCDA vesicles (TCDA/silica) (Fig. 1a, red left axis). These extreme changes in surface charge are indicative of changes to the surface chemistry; furthermore, they show that the layer was immobilized on the surface [32–34]. With the formation of the immobilized layer, the hydrodynamic diameter (HDD) of the vesicles increased from approximately 218 nm to 280 nm, indicating that the layer grew only at the surfaces of single vesicles (Fig. 1a, blue right axis and Additional file 1: Figure S2). It was also shown that the silica armor was securely attached to the TCDA vesicle surface. These vesicles had different chemical profiles. TCDA vesicles showed unique Fourier transform infrared spectroscopy (FTIR) peaks; methylene peaks at 2921 cm^−1^, and 2847 cm^−1^, and C = O stretching at 1693 cm^−1^ (Fig. 1b and Additional file 1: Table S1) [5]. PAH was immobilized on the TCDA vesicles by the formation of ionic bonds between carboxylates and NH3^+^ groups on the surface; the presence of peaks at approximately 3216 cm^−1^ (broad peak) for NH stretching, 1640 cm^−1^ for NH3^+^ asymmetric deformation, and at 1527 cm^−1^ for carboxylate asymmetric stretching are indicative of these ionic bonds [35, 36]. When silica armor forms on the vesicles, a Si–O–Si stretching peak can be observed at 1077 cm^−1^, indicating that the armor was well combined with the TCDA vesicles. [37]

The HDD of monomeric TCDA vesicles and TCDA/PAH in an aqueous medium was approximately 218–237 nm, whereas, as observed in TEM analysis (Additional file 1: Figure S3), the vesicles lacking the silica armor lost their original structure under dried state; hence, deformed to reduce their size range 20 to 70 nm. In contrast, the TCDA/silica vesicles maintained their structure sized at approximately 180 nm with the silica armor under vacuum conditions (Figs. 1c,d and Additional file 1: Figures S4 and S5). Because of the hard structure of the silica armor, which acted as a protective rind on the TCDA vesicles, the structural stability of the monomeric TCDA vesicles was enhanced under harsh environmental conditions. The area of the silica nanoparticles was determined from TEM images and their diameter was calculated (Fig. 1e). The TCDA/silica contained densely packed spherical silica nanoparticles with an average diameter of 4.7 ± 1.6 nm. TMOS has a high hydrolysis rate (k_TMOS_ = 48 L/mol• h); in contrast, tetraethylorthosilicate (TEOS) has a lower hydrolysis rate (k_TEOS_ = 0.37 L/mol h) in acidic solution; consequently, hydrolysis was completed within several minutes [16, 38]. Thus, the hydrolysis and nucleation of TMOS progressed quickly under the provided synthetic conditions and small silica nanoparticles were synthesized on the TCDA vesicles.

**Fig. 2:**
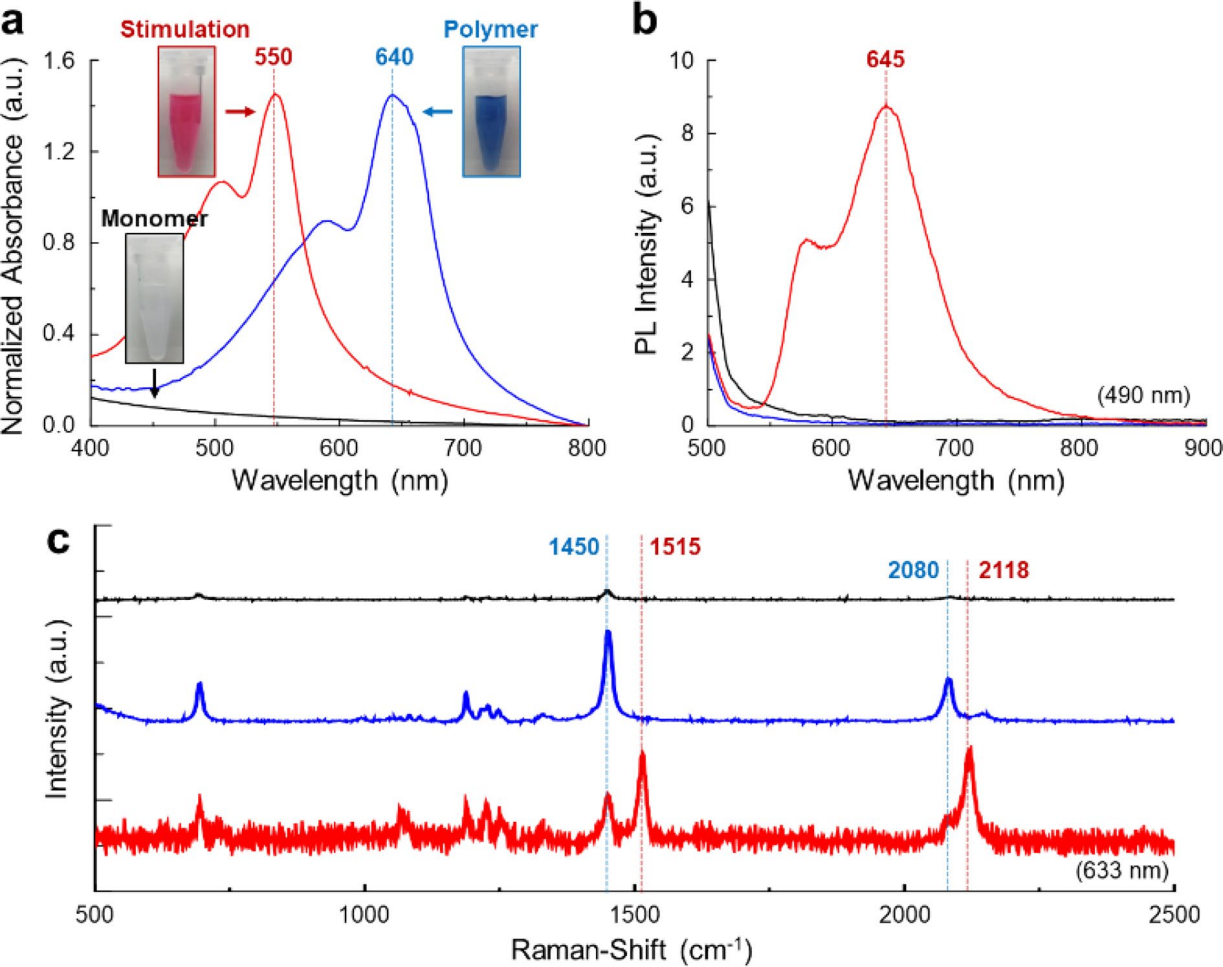
**a** Visible, **b** PL, and **c** Raman-shift spectra of TCDA/silica. Inset images of **a**: TCDA/silica with phases in deionized water. (Black line and box: monomeric phase, blue line and box: polymeric phase, and red line and box: thermal stimulated phase (~ 110 °C))

After surface modification, the stability of the silica-armored TCDA vesicles was confirmed by optical measurements. Once the DA structure was stable, the vesicles could be polymerized and stimulated to possess unique optical properties in terms of colorimetric response, visible-light absorption, photoluminescence (PL), and shifted peaks in their Raman spectra (Additional file 1: Figure S6) [5, 6]. The TCDA/silica clearly showed differentiated colors at each phase: white, blue, and red for the monomeric, polymeric, and stimulated phases, respectively (here, thermal stress of ~ 110 °C was used as the stimulus) (inset images in Fig. 2a). The monomeric phase did not have a specific absorption spectrum; however, the polymeric phase showed absorption peaks at 640 nm (main) and 590 nm (shoulder) (Fig. 2a). There was no change in the sizes of the TCDA/silica vesicles either before or after polymerization (Additional file 1: Figure S7). After stimulation, these peak positions shifted to 550 nm and 505 nm and a strong PL spectrum at ~ 645 nm was observed upon excitation at 490 nm (Fig. 2b). After polymerization, a strong Raman-shifted peak, arising from the resonance Raman phenomena of the C=C bond, was observed at 1450 cm^−1^; this bond was created by the 1,4-addition polymerization of the DA monomers (Fig. 2c) [4, 6]. Furthermore, the molecular structure changed upon stimulation, with peaks arising from C=C bonds moving to 1515 cm^−1^. Additionally, a C≡C bond peak was observed at 2080 cm^−1^ and moved to 2118 cm^−1^ upon stimulation. Consequently, we observed that the structural and optical properties of TCDA vesicles remained stable throughout the various modification steps.

Furthermore, the monomeric TCDA vesicles with silica armor stored in water at room temperature in the dark for two weeks showed enhanced dispersion stability compared to those without the armor. After two weeks, the stored monomeric TCDA vesicles had bulk-sized particles (> 5 μm). Monomeric TCDA vesicles could be easily altered, resulting in fusion and aggregation, and the ultimate formation of large particles. In contrast, TCDA/silica maintained its original structure owing to the high chemical stability of the silica on the surface.

**Fig. 3:**
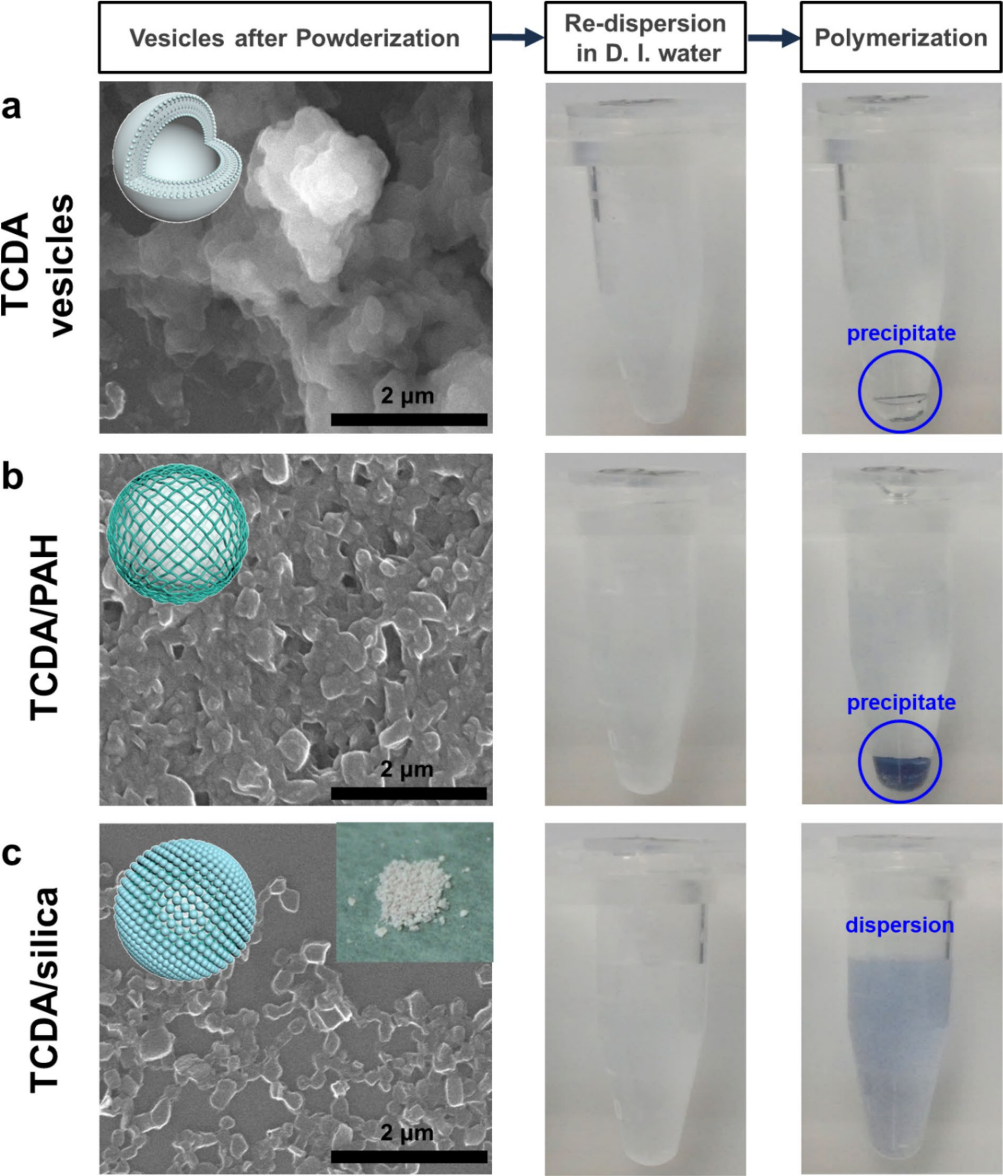
Redispersibility of TCDA vesicles with layer encapsulation. SEM images of monomeric **a** TCDA vesicles, **b** TCDA/PAH, and **c** TCDA/silica after powderization (Inset image of **c**: TCDA/silica powder), and photo images of the sonicated monomeric powders into deionized water and its polymeric phase

**Fig. 4:**
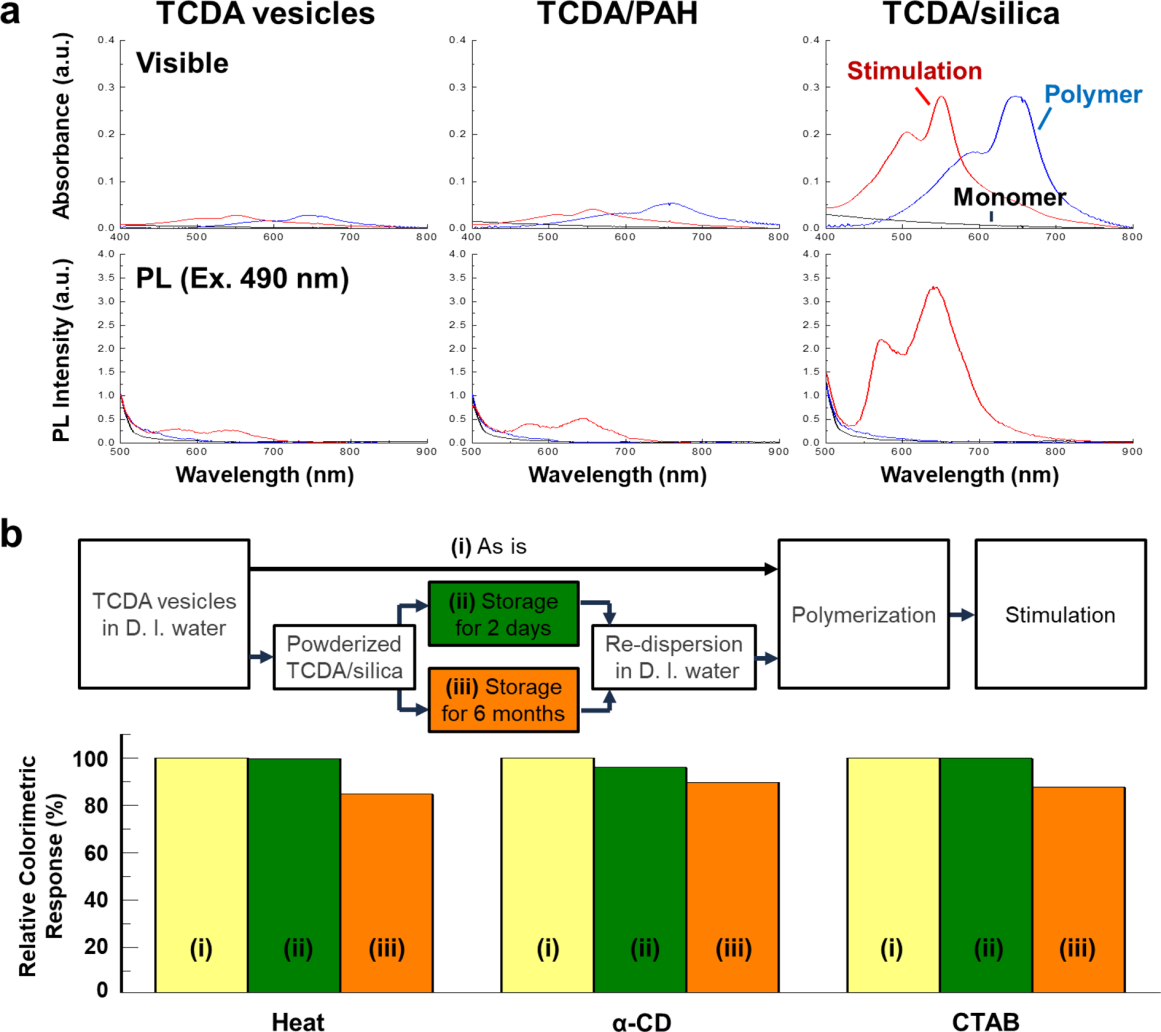
**a** Visible and PL spectra of redispersed monomeric TCDA vesicles into deionized water with layer encapsulation and phases (black line: monomeric phase, blue line: polymeric phase, and red line: thermal stimulated phase (~ 110 °C)), **b** Sustainable sensing response of TCDA vesicles with layer encapsulation. Relative CR values of reactivity test of (i) unstored TCDA vesicles and redispersed TCDA/silica after (ii) 2 days and (iii) 6 months

The silica armor also protected monomeric TCDA vesicles in the dried powder state. The dried monomeric TCDA vesicles could not be redispersed in water, implying that the vesicle structure was disrupted by the drying process, thereby preventing polymerization (Figs. 3a and 4a). When monomeric TCDA vesicles were encapsulated with PAH, the vesicles in the dried state after powderization partially formed aggregates; hence, the TCDA/PAH was poorly redispersible. Despite this, the bulk structures of TCDA/PAH exhibited optical properties after polymerization and stimulation (Figs. 3b and 4a). In contrast, the TCDA/silica maintained their structures even after powderization. High redispersibility in water, strongly visible PL intensity, and a Raman-shifted peak corresponding to the phases were observed (Figs. 3c and Additional file 1: Figure S8). The formation of silica armor prevented the aggregation of monomeric TCDA vesicles and rendered them chemically and structurally stable. Moreover, it prevented disruption of the vesicle structure under drying conditions and induced high redispersibility of TCDA/silica in water (Additional file 1: Figure S9). Therefore, it can be stated that the stability of the TCDA vesicles was greatly enhanced by encapsulation in the silica armor, dramatically increasing colloidal dispersibility.

To confirm the stimulus-driven response of the TCDA/silica over long storage periods, we monitored the optical properties after exposure to thermal and chemical stimuli, such as heating at ~ 110 °C and exposure to α-cyclodextrin (α-CD) and cetyltrimethylammonium bromide (CTAB) [39, 40]. Because thermal energy causes partial distortion of the polymer backbone and reduces the conjugation length, the optical properties change [5]. The chemical stimuli directly influence the head groups and conjugated domain of PDA, subsequently inducing optical responses. The colorimetric response (CR) depended on the changes in the conjugation lengths of the π-electrons along the PDA backbone due to the stimuli. For example, a red color (approximately 12 nm) occurs upon heating and exposure to α-CD and a yellow color (approximately 8 nm) occurs upon exposure to CTAB [41, 42]. These colorimetric variations can be quantitatively measured using CR values. [7, 8] After stimulation, polymeric TCDA vesicles showed colorimetric changes from blue to red or yellow. The colorimetric response (CR) values of the TCDA vesicles and redistributed TCDA/silica powders according to the storage period were calculated (Fig. 4b and Additional file 1: Table S2). After storage for two days, the change in the CR value of the TCDA/silica powder was similar to that of the unstored TCDA vesicles. Furthermore, redispersed TCDA/silica powder stored for six months clearly showed a colorimetric change when stimulated. The relative CR values were 84.8% (heat), 89.8% (α-CD), and 87.6% (CTAB) compared to TCDA vesicles, proving that the colorimetric response of the TCDA/silica powder could be maintained for a long period of approximately 6 months, which we believe is a phenomenal result that has not previously been achieved. The slight decrease in the CR values by approximately 10–15% compared to those of fresh polymeric TCDA vesicles could be due to gradual oxidation [43] of PDA in the silica armor over a long period. We demonstrated that the silica armor did not adversely affect the optical function of the TCDA vesicles but instead resulted in long-term sustainability. The stimulating agents could diffuse easily through the nanopores of the silica layer into the vesicles; each elicited a unique colorimetric response upon interaction with PDA. This indicates that the silica armor serves only to protect the TCDA vesicles and does not affect their optical properties.

**Fig. 5:**
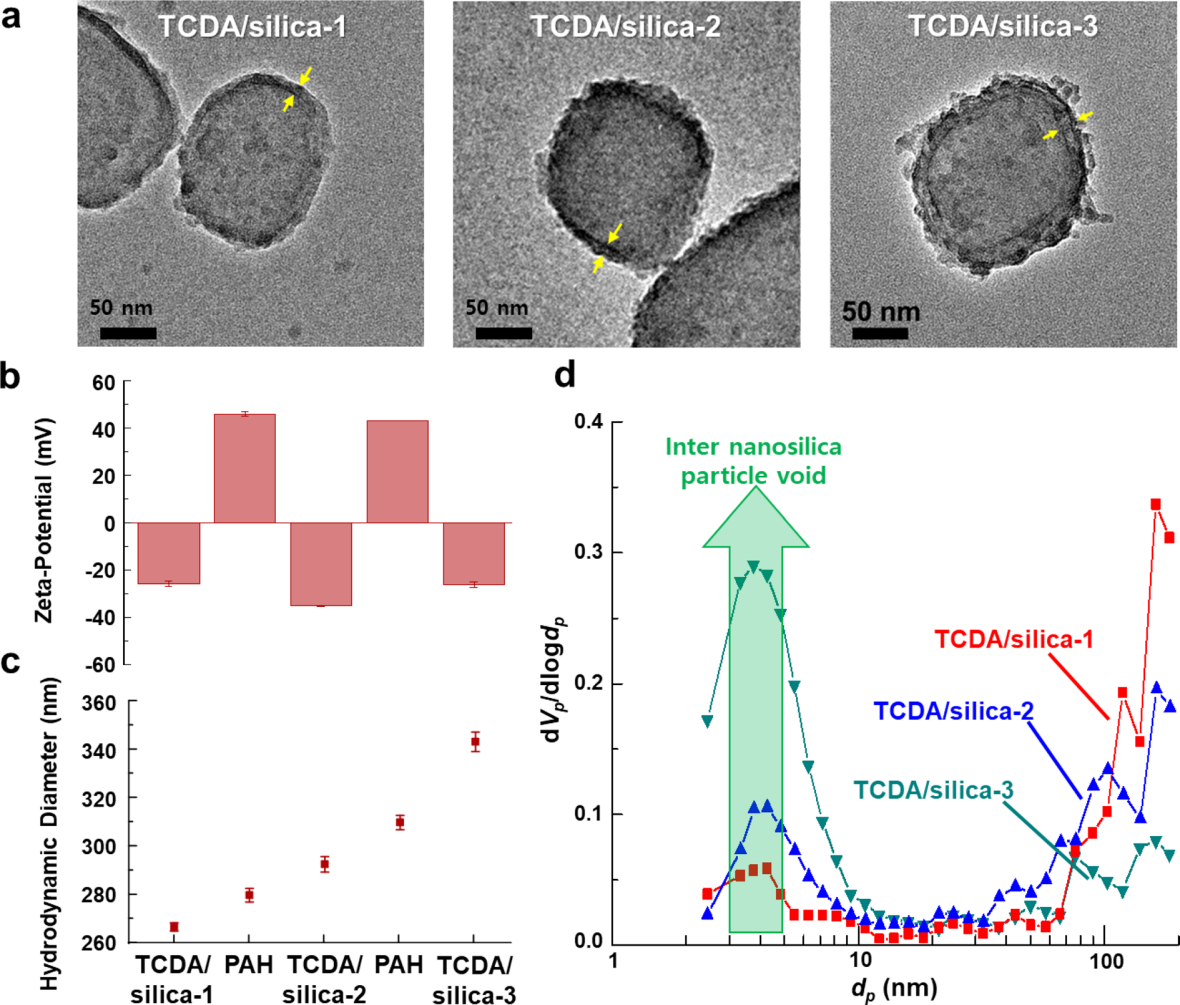
**a** TEM images, **b** Surface charge and **c** HDD of monomeric TCDA vesicles with repeated silica armor membranes. **d** Brunauer–Emmett–Teller (BET) analysis results of the silica armor membranes on monomeric TCDA vesicles. The n value of PCDA/silica-n indicates the number of silica armor membranes

Furthermore, by repeating the silica armor membrane synthesis using the LbL approach, many layers of the silica armor membrane could be continuously built on the surface of the vesicles. By repetitive surface modification and silica hydrolysis on the TCDA vesicles, approximately 5 nm spherical silica nanoparticles could be densely coated on the TCDA vesicle surfaces. The silica nanoparticles could be continuously grown using our method and with a number of repetitions, the thickness of the silica armor membrane was increased to approximately 5.0 ± 2.5 (1 repetition, TCDA/silica-1), 8.0 ± 3.3 (2 repetitions, TCDA/silica-2) and 12.2 ± 3.8 nm (3 repetitions, TCDA/silica-3) (Figs. 5a and Additional file 1: Figure S10). Moreover, it was observed that the surface charge of the vesicles depended on the surface chemistry and treatment steps and the synthesized vesicles changed from negative (due to carboxylic groups) to positive upon coating with PAH (Fig. 5b). Furthermore, the addition of a silica nanoparticle layer causes a subsequent reversal of the charge from positive to negative. These changes were repeatedly observed when the surface charge of the vesicles was approximately − 37 mV to + 57 mV. HDD analysis revealed that the TCDA vesicles gradually increased in size from approximately 220 nm to 340 nm (Fig. 5c). These results also indicate that the silica armor membranes were uniformly grown on the surface of the TCDA vesicles and that the TCDA/silica was highly dispersible in water.

To confirm the internal structures of the silica armor membranes, the average pore size and surface area of TCDA/silica were measured using Brunauer–Emmett–Teller (BET) analysis (Fig. 5d). The silica nanoparticles constituting the membrane had pores through which stimuli passed; consequently, the silica armor membranes enabled size-selective permeability for the stimuli to act on the TCDA vesicles. When the silica armor membranes were formed, the average pore size was 33.58 nm for the dried TCDA/silica-1, which decreased to 12.97 nm for TCDA/silica-2, and further to 4.30 nm for TCDA/silica-3. This was due to an increase in the number of voids between the nanosilica particles. The concomitant increase in the surface area ranged from 10.06 m^2^/g to 182.73 m^2^/g while increasing the amount of silica nanoparticles on the membrane. Thus, when the thickness of the silica armor membrane was increased by the repetitive coating process, the silica nanoparticles were observed to overlap with one another on the surface, increasing the paths through which the stimuli passed, thus making it difficult to reach the inside. These results indicate that the present method used to form the silica armor membranes also controlled their porosities. Therefore, this method can provide size-selective recognition for TCDA vesicles.

Next, using the above structural properties, we confirmed the size-selective recognition of TCDA vesicles with silica armor membranes using stimuli of different sizes. When stimulus size is sufficiently smaller than the average pore size, it can diffuse through the membrane and stimulate the TCDA vesicles. We investigated the recognition of the TCDA vesicles with 10 mM solutions of differently sized molecules, all of which formed their own specific structures by transforming their structures to a low-energy state: sodium hydroxide (NaOH, breaking down to ions of ~ 0.15 nm), cetyltrimethylammonium bromide (CTAB, forming micelles of ~ 5 nm), α-cyclodextrin (α-CD, forming self-aggregates of ~ 65 nm), and γ-cyclodextrin (γ-CD, forming self-aggregates of ~ 77 nm) [44–47]. The first three stimuli, except for γ-CD, directly changed the conjugation length of PDA by breaking the hydrogen bonds through electrostatic attraction forces and hydrophobic interactions. These stimuli subsequently induced optical responses that turned the blue to red or orange phases [39, 48, 49]. In the case of γ-CD, there was no optical response with PDA [50–52]. These optical responses could be quantitatively measured using colorimetric response (CR) analysis. [53]

**Fig. 6:**
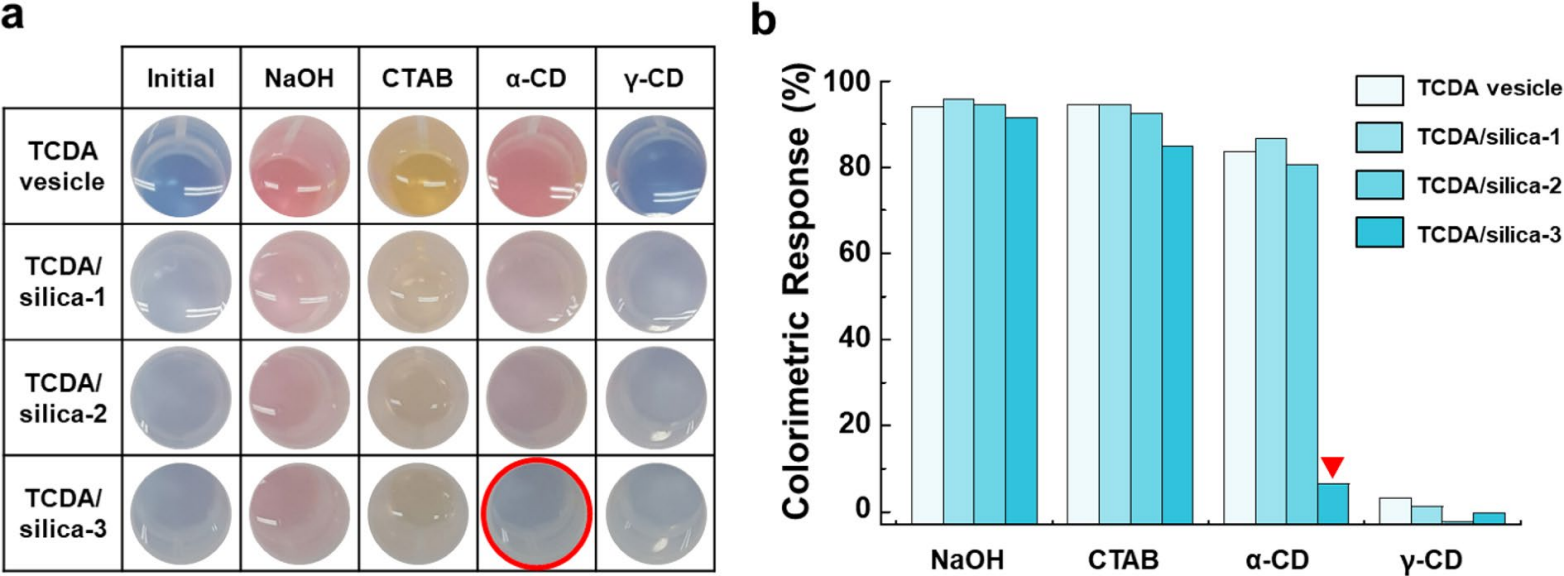
Size-selective recognition of TCDA vesicles in silica armor membranes. **a** Photo-images and **b** colorimetric response values of TCDA vesicle in silica membrane with 10 mM of chemical stimuli. The CR value of PCDA/silica-3 lowered significantly in response to α-CD (red highlight). The n value of PCDA/silica-n indicates the number of silica armor membranes

The polymerized TCDA vesicles had a blue color and a visible spectrum with a main peak at ~ 640 nm, whereas no PL signals were present (Figs. 6a, Additional file 1: Figure S11 and S12). The TCDA vesicles exhibited optical changes when exposed to the stimuli: color changes to red and orange, visible spectra changed to ~ 540 nm or ~ 500 nm, and PL was induced at ~ 570–650 nm. The CR values corresponding to the color changes were 94% for NaOH, 94% for CTAB, and 84% for α-CD (Fig. 6b). Because such optical responses are induced by changes in the conjugated length caused by exposure to stimuli, the changes are influenced by interactions between the PDA backbone and stimuli. For example, a shift from the blue (infinity conjugated length) to the red phase (conjugated length: ~ 12 nm) occurred upon exposure to NaOH and α-CD, and from the blue to the yellow phase (~ 8 nm) upon exposure to CTAB [41]. Therefore, polymerized TCDA vesicles changed to the red phase due to NaOH and α-CD and to the orange phase due to CTAB. However, in the case of γ-CD, colorimetric changes did not occur because it did not affect the conjugated length. The same optical responses were observed in silica-armored TCDA vesicles. NaOH and CTAB showed similar optical responses and CR values to those of the plain TCDA vesicles, regardless of the number of membranes because even though silica armor membranes had formed on the surface of the TCDA vesicles, these membranes still had many pores that were large enough for the stimuli to pass through. Thus, these stimuli could diffuse through the membrane, affecting TCDA vesicles at their cores. In contrast, α-CD showed different optical responses depending on the number of membranes. It diffused TCDA vesicles, causing a blue-to-red transition for TCDA/Silica-1 and 2, but not for TCDA/silica-3 because of the long narrow passages between the layers around the TCDA vesicles. This means that the number of silica layers required to maintain a sustainable response for α-CDs was less than three. In summary, the pores in the membrane were able to screen out large molecules, thereby enabling size-selective recognition of the TCDA vesicles. Therefore, using our silica armor membrane building method, we should control the number of silica membranes that can be applied to maintain a sustainable response from the TCDA vesicle sensor.

## Conclusions

In this study, we first formed a silica armor membrane to cover soft and delicate DA vesicles like an armor. Owing to the rigid silica armor structure, the stability of DA vesicles was significantly improved and the redispersion property was confirmed through powderization. The vesicles with the silica armor membrane sustained colorimetric response to stimuli by an average of 87.4% compared to unstored vesicles, proving that sensing performance was maintained even after long-term storage for 6 months; this phenomenal result had never before been achieved. Furthermore, the path of the stimuli passing between the silica nanoparticles in membrane became longer as the thickness of the membrane increased. the PDA vesicle with multilayers of silica armor membrane had size-selective recognition for screening out stimuli. Silica armor can be applied to various assembly structures of organic materials using the modified Stöber reaction and electrostatic LBL approach. We expect this technique to have a huge impact on the practical applications of organic material-based sensor devices and silica membrane technology.

## Supplementary Information

**Additional file 1: Figure S1**. Molecular structure of materials. **Figure S2**. Size distribution of monomeric TCDA vesicles with the layer. **Figure S3**. TEM images of monomeric TCDA vesicles and TCDA/PAH. **Figure S4**. TEM images of monomeric TCDA/silica. **Figure S5**. SEM image of monomeric TCDA/silica. **Figure S6**. (a) Photo image, (b) visible, (c) PL, and (d) Raman spectra of TCDA vesicles; monomeric phase (black line), polymeric phase (blue line) and stimulated phase (red line). **Figure S7**. (a) TEM and (b) SEM image of polymerized TCDA/silica, and EDS spectra of section shown by the white line in (a). **Figure S8**. Raman-shift spectra of redispersed TCDA/silica into deionized water. (black line: monomeric phase, blue line: polymeric phase, and red line: thermal stimulated phase (~ 110 oC)). **Figure S9**. Size distribution of redispersed monomeric TCDA/silica into deionized water. **Figure S10**. TEM images of TCDA/silica. **Figure S11**. Visible spectra of polymerized TCDA vesicles and TCDA/Silica particles with 10 mM of chemical stimuli. **Figure S12**. PL spectra of polymerized TCDA vesicles and TCDA/Silica particles with 10 mM of chemical stimuli (Excitation at 490 nm). **Table S1**. FT-IR peak assignment of Figure 1d. **Table S2**. Colorimetric response (CR) values of TCDA vesicles and redispersed TCDA/silica powder with storage period.

## Abbreviations

PDA: Polydiacetylene
DA: Diacetylene
LbL: Layer-by-layer
TCDA: 10,12-Tricosadiynoic acid
PAH: Polyallylamine hydrochloride
TMOS: Tetramethylorthosilicate
HDD: Hydrodynamic diameter
FTIR: Fourier-transform infrared spectroscopy
TEM: Transmission electron microscopy
TEOS: Tetraethylorthosilicate
CTAB: Cetyltrimethylammonium bromide
α-CD: α-Cyclodextrin
CR: Colorimetric response
BET: Brunauer Emmett –Teller
