# Supplementary material for: Sustainable colorimetric/luminescent sensors enabled by armored lipid nanoparticles
Source: Nano Converg. 2022 Sep 30;9:42. doi: 10.1186/s40580-022-00335-5 (PMC9525522; doi:10.1186/s40580-022-00335-5)
Supplement: Supplementary file 1 — Additional file 1: Figure S1. Molecular structure of materials. Figure S2. Size distribution of monomeric TCDA vesicles with the layer. Figure S3. TEM images of monomeric TCDA vesicles and TCDA/PAH. Figure S4. TEM images of monomeric TCDA/silica. Figure S5. SEM image of monomeric TCDA/silica. Figure S6. (a) Photo image, (b) visible, (c) PL, and (d) Raman spectra of TCDA vesicles; monomeric phase (black line), polymeric phase (blue line) and stimulated phase (red line). Figure S7. (a) TEM and (b) SEM image of polymerized TCDA/silica, and EDS spectra of section shown by the white line in (a). Figure S8. Raman-shift spectra of redispersed TCDA/silica into deionized water. (black line: monomeric phase, blue line: polymeric phase, and red line: thermal stimulated phase (~ 110 oC)). Figure S9. Size distribution of redispersed monomeric TCDA/silica into deionized water. Figure S10. TEM images of TCDA/silica. Figure S11. Visible spectra of polymerized TCDA vesicles and TCDA/Silica particles with 10 mM of chemical stimuli. Figure S12. PL spectra of polymerized TCDA vesicles and TCDA/Silica particles with 10 mM of chemical stimuli (Excitation at 490 nm). Table S1. FT-IR peak assignment of Figure 1d. Table S2. Colorimetric response (CR) values of TCDA vesicles and redispersed TCDA/silica powder with storage period. [file 40580_2022_335_MOESM1_ESM.docx]

Nano Convergence

Supporting Information

**Sustainable Colorimetric/Luminescent Sensors**

**Enabled by Armored Lipid Nanoparticles**

Jinkyu Roh^1^, Yong Ho Cho^2^, and Dong June Ahn^*,1,2^

^1^Department of Chemical and Biological Engineering, Korea University, Seoul 02841, Republic of South Korea.

^2^KU-KIST Graduate School of Converging Science and Technology, Korea University, Seoul 02841, Republic of South Korea.


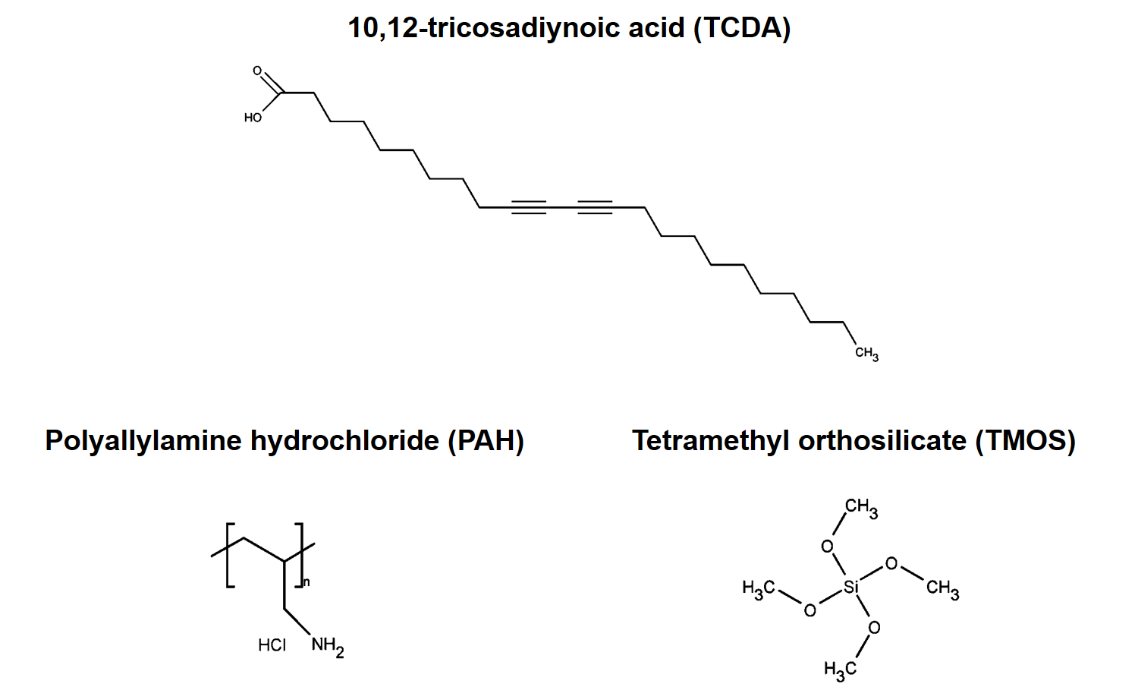


**Figure S1.** Molecular structure of materials.


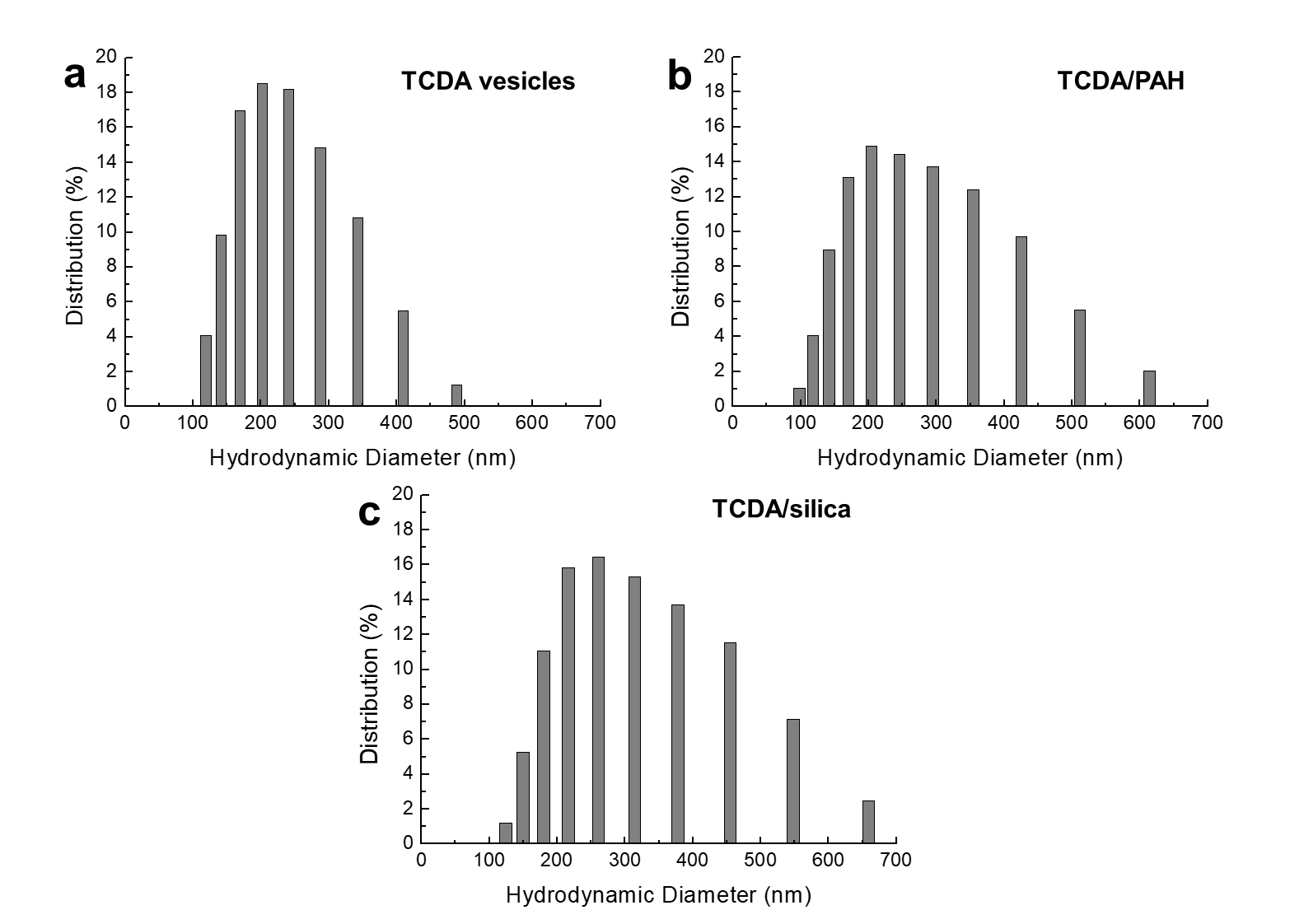


**Figure S2.** Size distribution of monomeric TCDA vesicles with the layer.

**Table S1.** FT-IR peak assignment of Figure 1d.


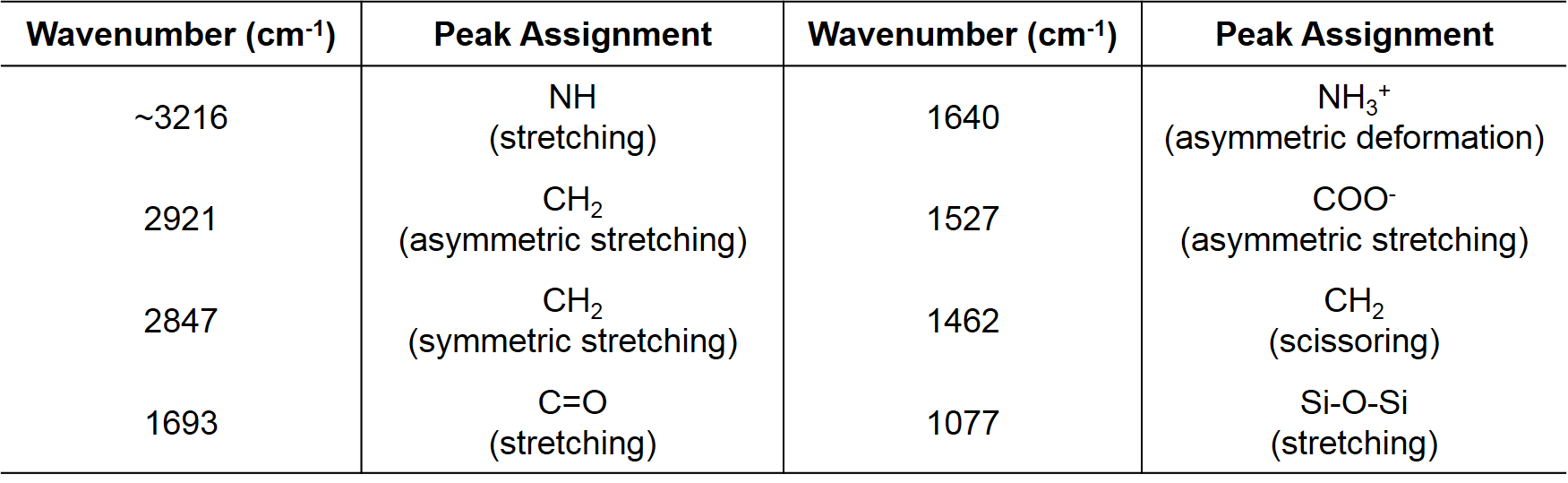


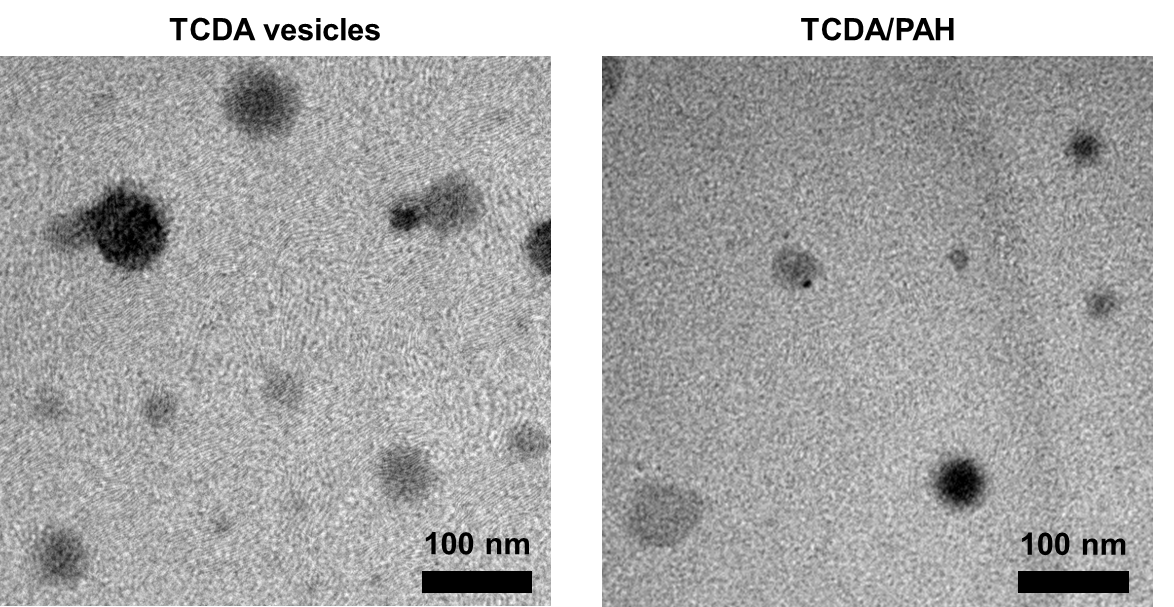


**Figure S3.** TEM images of monomeric TCDA vesicles and TCDA/PAH


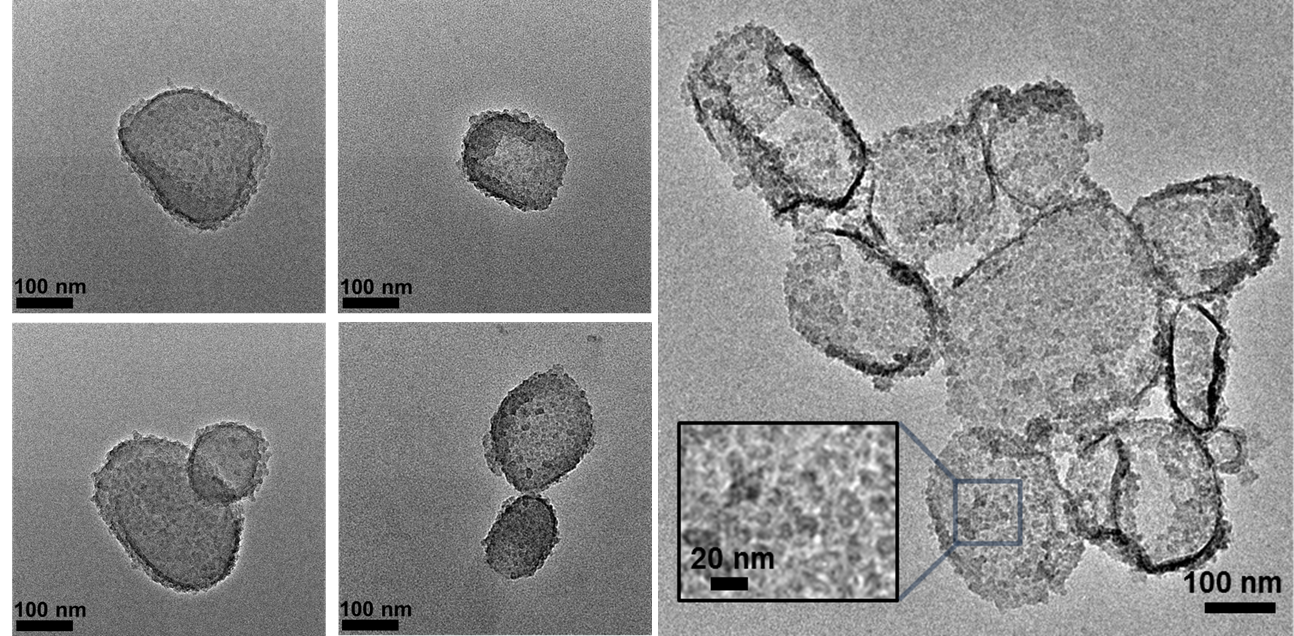


**Figure S4.** TEM images of monomeric TCDA/silica.


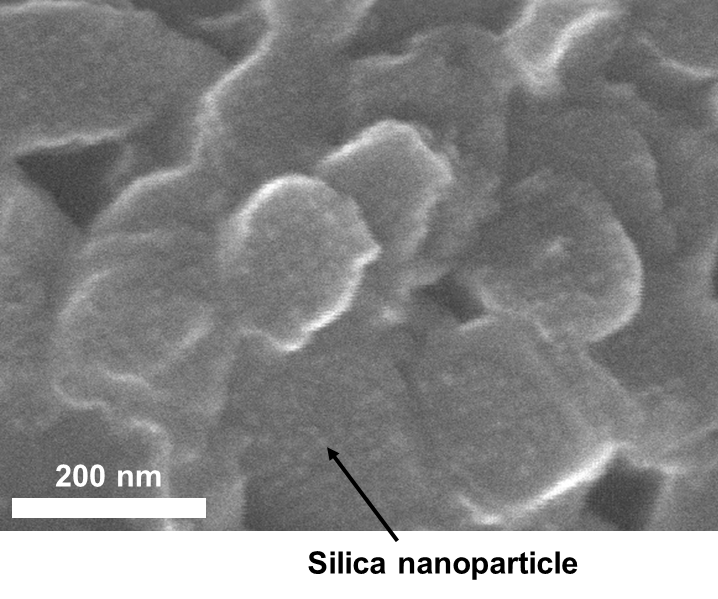


Figure S5. SEM image of monomeric TCDA/silica.
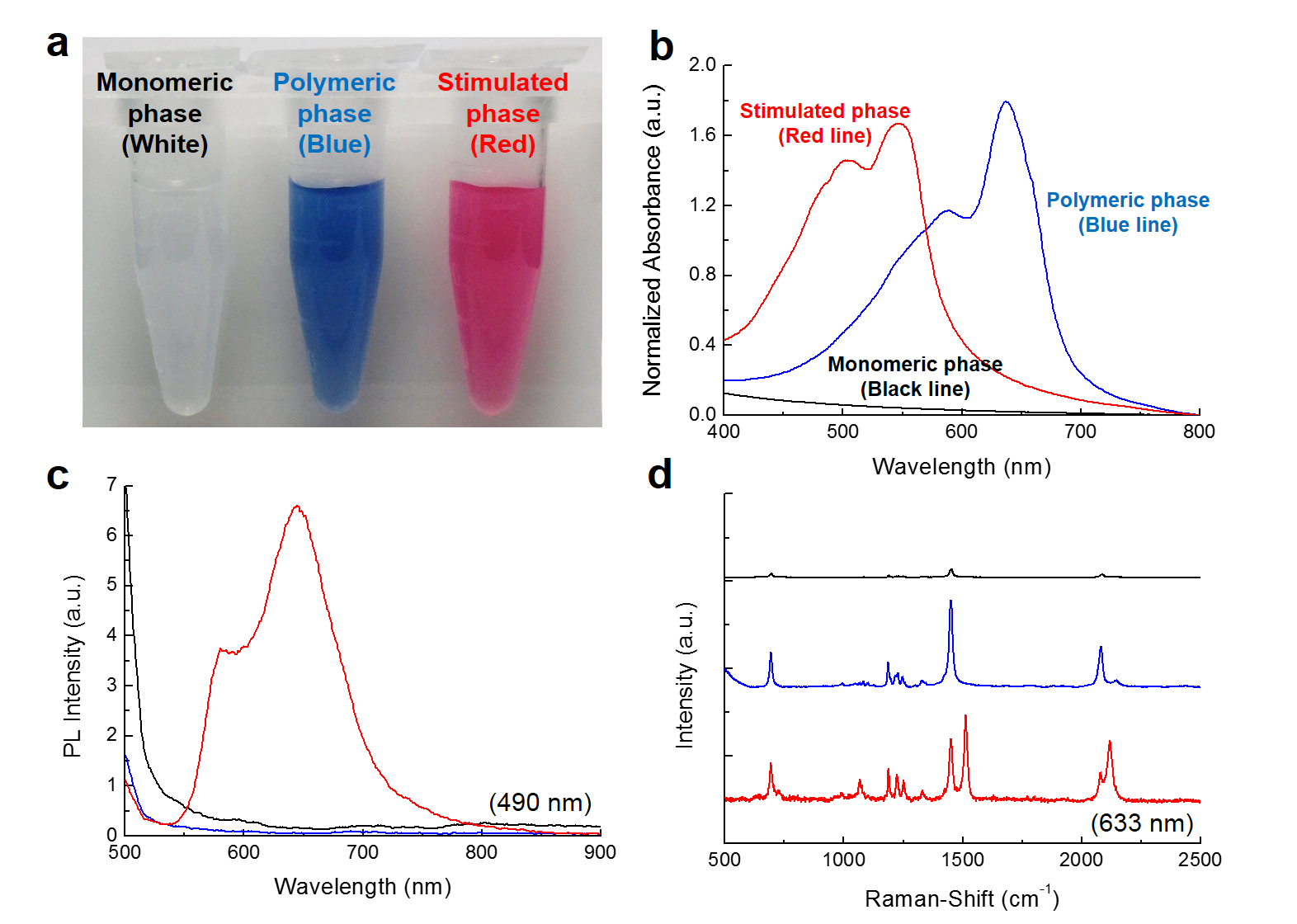


Figure S6. (a) Photo image, (b) visible, (c) PL, and (d) Raman spectra of TCDA vesicles; monomeric phase (black line), polymeric phase (blue line) and stimulated phase (red line).


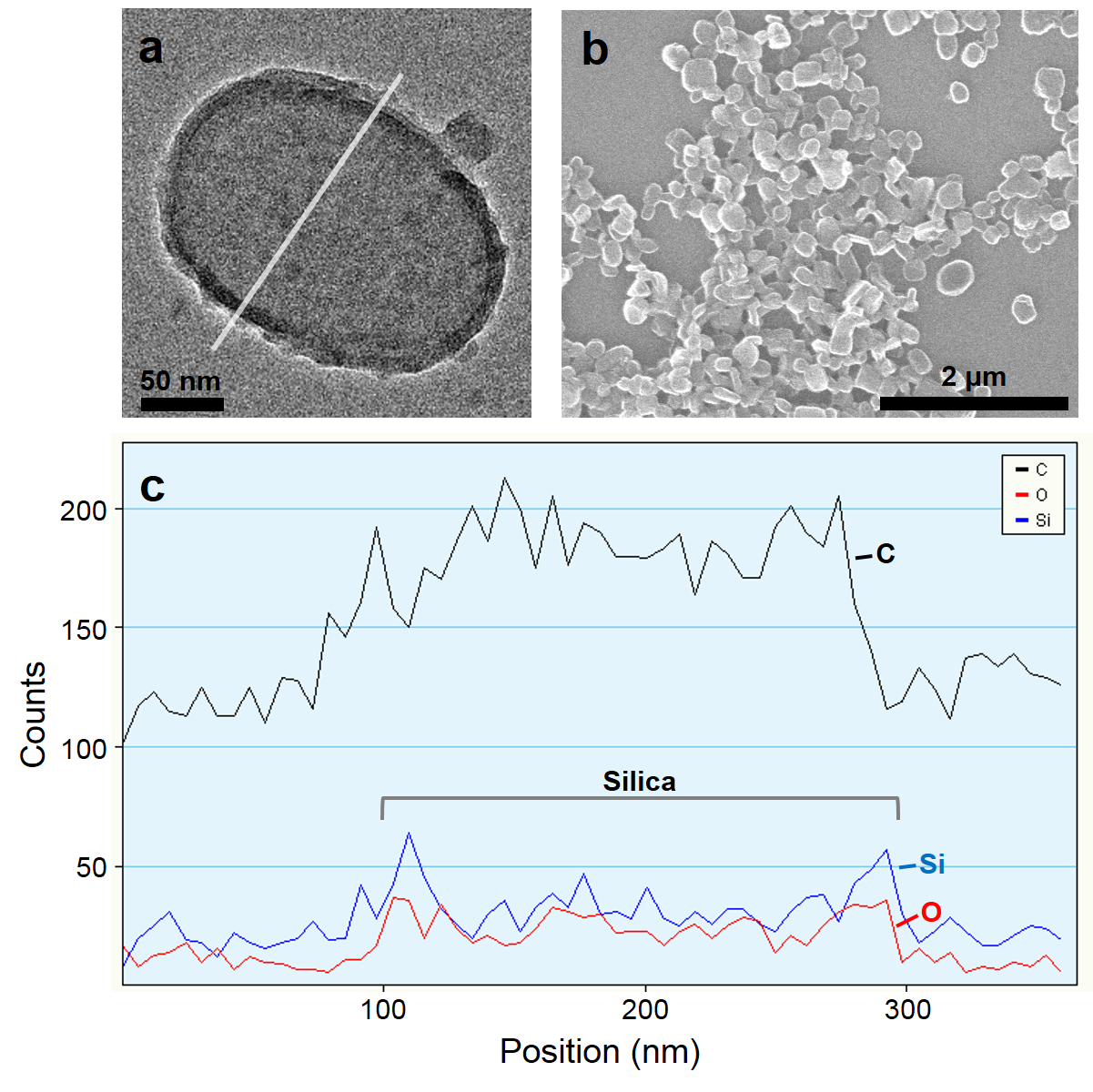


**Figure S7.** (a) TEM and (b) SEM image of polymerized TCDA/silica, and EDS spectra of section shown by the white line in (a).


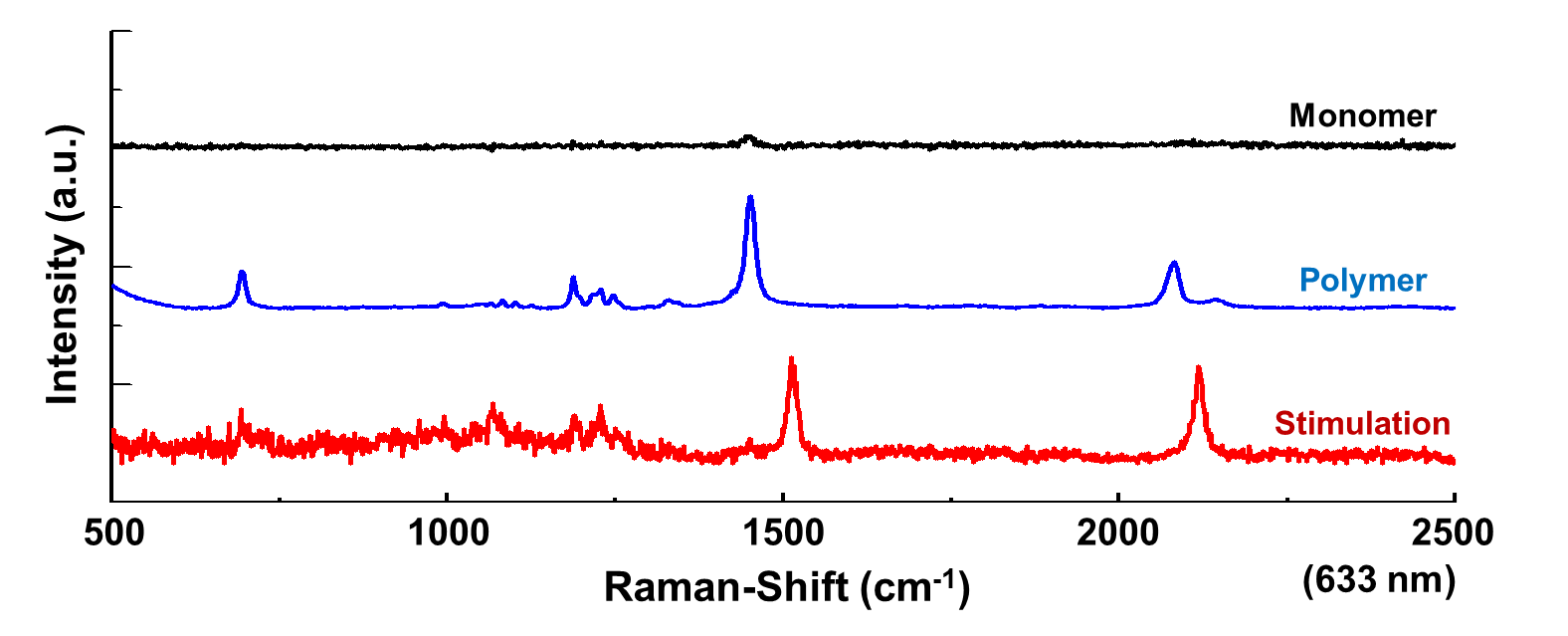


**Figure S8.** Raman-shift spectra of redispersed TCDA/silica into deionized water. (black line: monomeric phase, blue line: polymeric phase, and red line: thermal stimulated phase (~ 110 ^o^C))

**
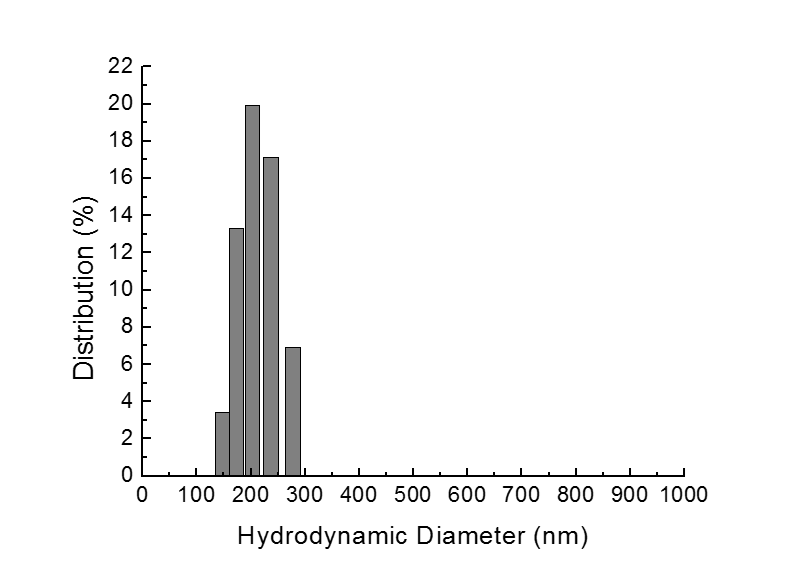
**

**Figure S9.** Size distribution of redispersed monomeric TCDA/silica into deionized water.

**Table S2.** Colorimetric response (CR) values of TCDA vesicles and redispersed TCDA/silica powder with storage period.


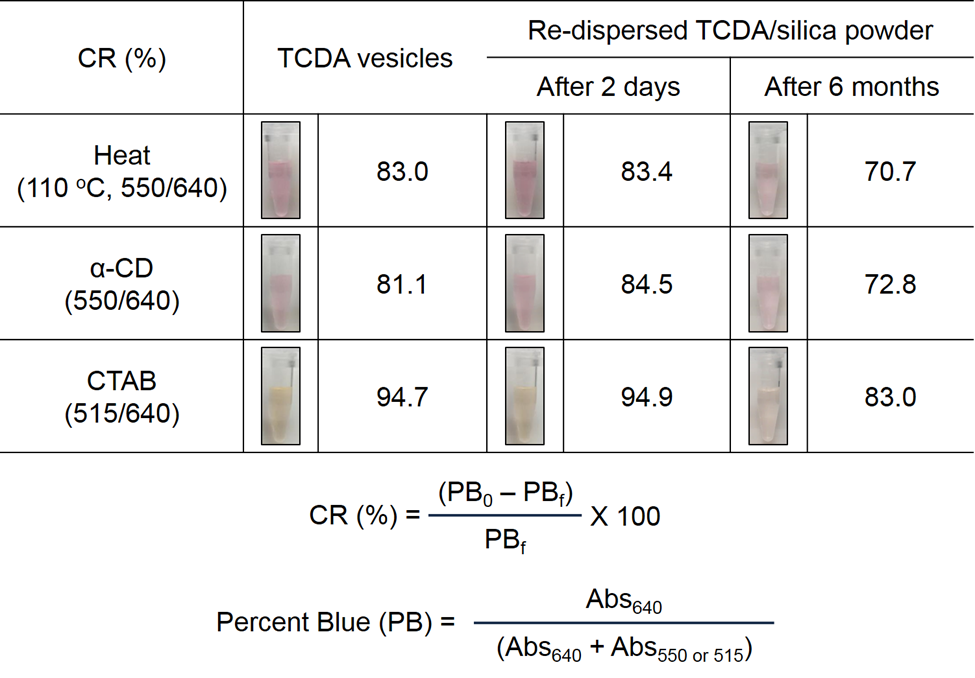


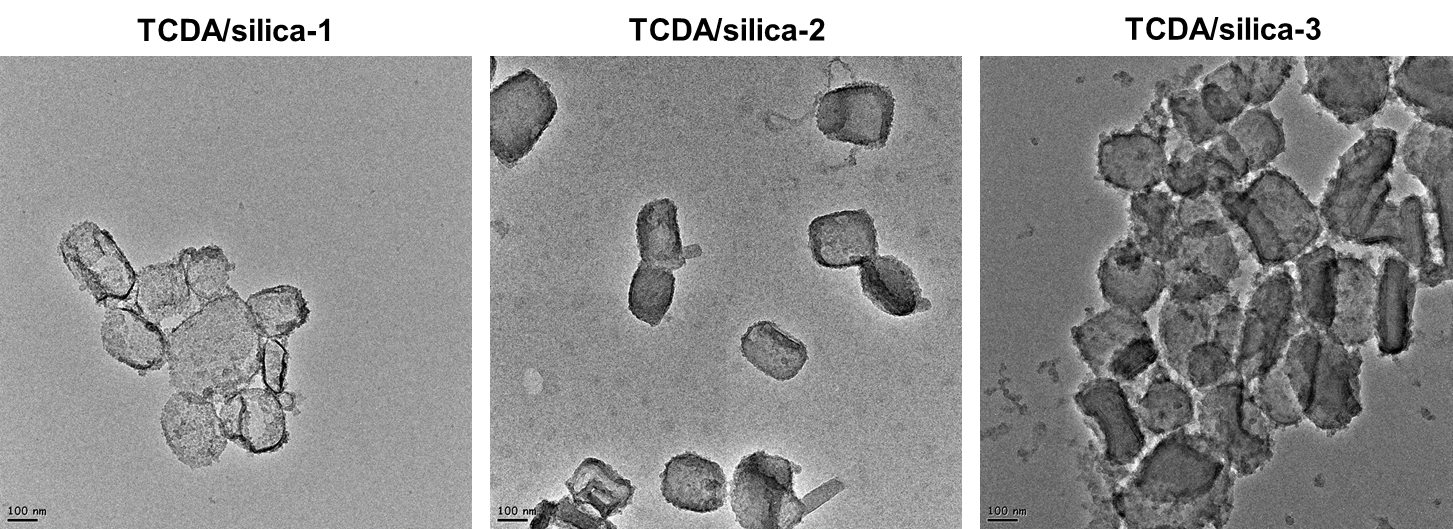


**Figure S10.** TEM images of TCDA/silica.


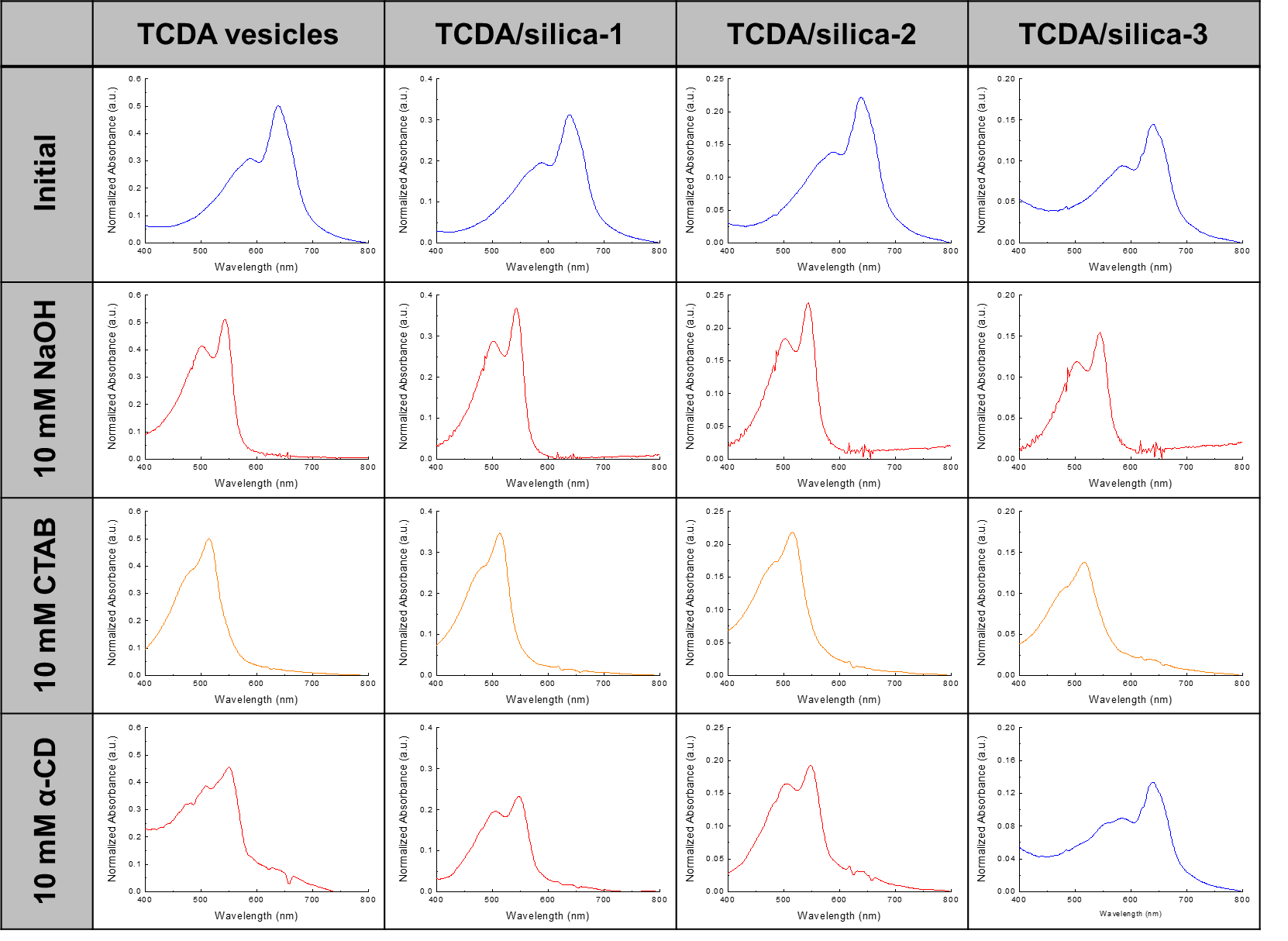


**Figure S11**. Visible spectra of polymerized TCDA vesicles and TCDA/Silica particles with 10 mM of chemical stimuli.

**
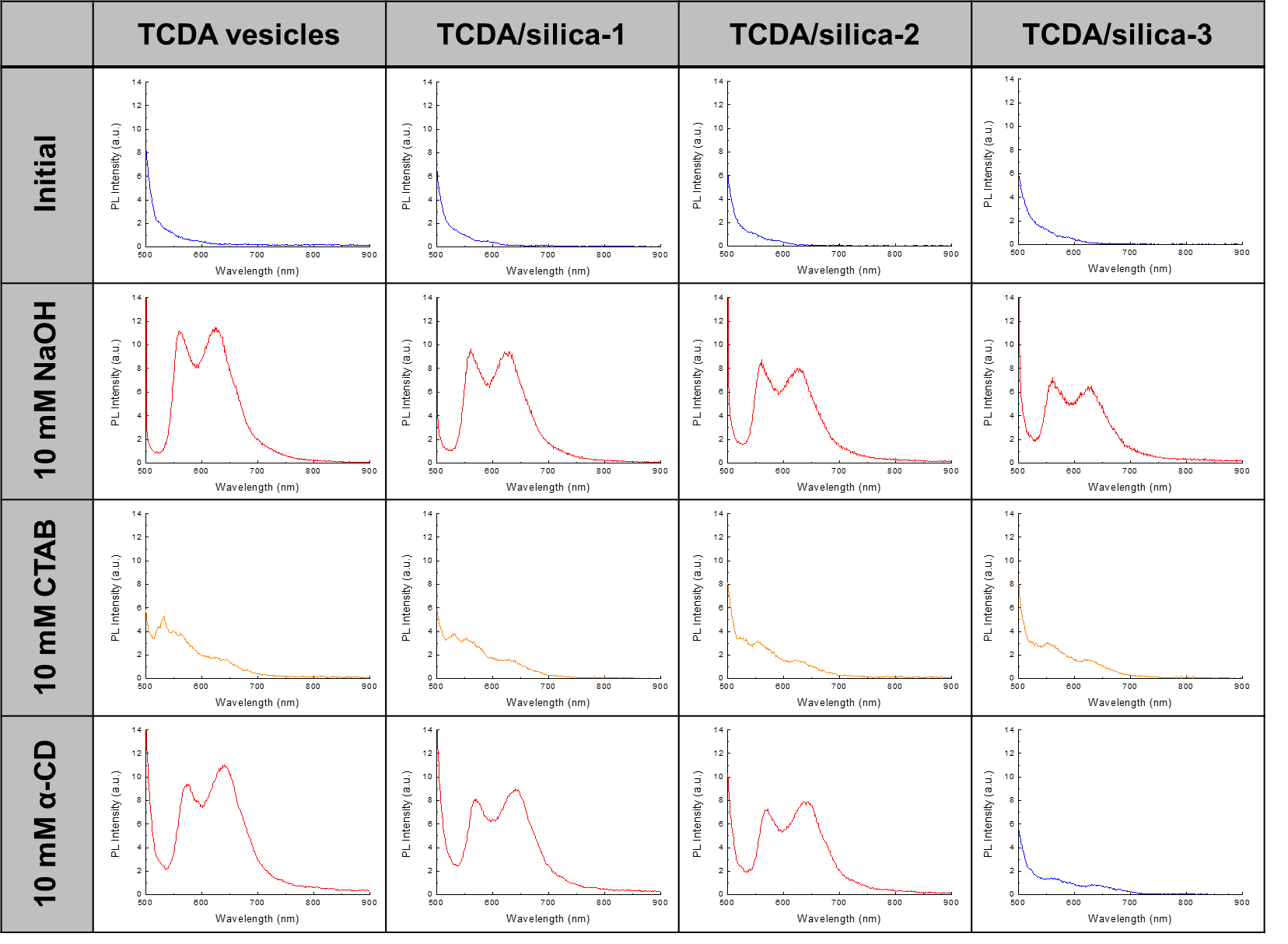
**

**Figure S12**. PL spectra of polymerized TCDA vesicles and TCDA/Silica particles with 10 mM of chemical stimuli (Excitation at 490 nm).
